# GAIA: a gram-based interaction analysis tool – an approach for identifying interacting domains in yeast

**DOI:** 10.1186/1471-2105-10-S1-S60

**Published:** 2009-01-30

**Authors:** Kelvin X Zhang, BF Francis Ouellette

**Affiliations:** 1 Graduate Program in Bioinformatics, University of British Columbia, Vancouver, British Columbia, V6T 1Z4, Canada; 2 Ontario Institute for Cancer Research, MaRS Centre, South Tower, 101 College Street, Toronto, Ontario, M5G 0A3, Canada

## Abstract

**Background:**

Protein-Protein Interactions (PPIs) play important roles in many biological functions. Protein domains, which are defined as independently folding structural blocks of proteins, physically interact with each other to perform these biological functions. Therefore, the identification of Domain-Domain Interactions (DDIs) is of great biological interests because it is generally accepted that PPIs are mediated by DDIs. As a result, much effort has been put on the prediction of domain pair interactions based on computational methods. Many DDI prediction tools using PPIs network and domain evolution information have been reported. However, tools that combine the primary sequences, domain annotations, and structural annotations of proteins have not been evaluated before.

**Results:**

In this study, we report a novel approach called Gram-bAsed Interaction Analysis (GAIA). GAIA extracts peptide segments that are composed of fixed length of continuous amino acids, called n-grams (where n is the number of amino acids), from the annotated domain and DDI data set in *Saccharomyces cerevisiae *(budding yeast) and identifies a list of n-grams that may contribute to DDIs and PPIs based on the frequencies of their appearance. GAIA also reports the coordinate position of gram pairs on each interacting domain pair. We demonstrate that our approach improves on other DDI prediction approaches when tested against a gold-standard data set and achieves a true positive rate of 82% and a false positive rate of 21%. We also identify a list of 4-gram pairs that are significantly over-represented in the DDI data set and may mediate PPIs.

**Conclusion:**

GAIA represents a novel and reliable way to predict DDIs that mediate PPIs. Our results, which show the localizations of interacting grams/hotspots, provide testable hypotheses for experimental validation. Complemented with other prediction methods, this study will allow us to elucidate the interactome of cells.

## Background

Biological functions of cells are determined by strict regulations of molecular interactions of proteins, lipids, carbohydrates and nuclear acids both temporally and spatially. Protein-Protein Interactions (PPIs) play important roles in all biological functions from enzyme catalysis, signal transduction, as well as many structural functions. Owing to advances in large-scale techniques such as the yeast two-hybrid system and affinity purification followed by mass spectrometry, interactomes of several model organisms such as *Saccharomyces cerevisiae *[1-6], *Drosophila melanogaster *[7,8] and *Caenorhabditis elegans *[9] have recently been extensively studied. While such large-scale interaction data sets provide tremendous opportunities for data exploration although there are limitations: 1) the experimental techniques for detecting PPIs are time-consuming, costly and labour intensive; 2) the quality of certain datasets is uneven; and 3) technical limitations such as the requirement to tag proteins of interest still exist. It has been widely accepted that some proteins interact with each other through interactions between their domains, which are defined as independently structural and/or functional blocks of proteins. For example, some cytoskeletal proteins interact with actin because of the interaction between their gelsolin repeat domains [10]. It has also been reported that sets of conserved residues within the WW domains can bind to proline-rich peptides [11]. Therefore, the identification of DDIs can potentially shred light on the mechanism underlying PPIs. Unfortunately, identifying neither DDIs nor PPIs through experimental approaches is trivial. As a complementary alternative, computational approaches that identify DDIs have been studied intensively for years yielding some interesting results.

The currently available computational DDI prediction approaches can be categorized as follows: 1) Association-based approaches where each DDI is scored by the association of the number of interacting domain pairs between interacting protein pairs and non-interacting protein pairs. These methods, however, only compute each DDI locally without considering the information of other DDIs between protein pairs [12-14]. Deng *et al. *proposed an optimized approach, maximum likelihood estimation (MLE), which globally calculates the probabilities of interaction between two domains using the expectation-maximization (EM) algorithm [15]. 2) Pattern-based approaches where the domain interaction pattern of each interacting protein pair is utilized to predict DDIs by applying machine learning approaches such as clustering algorithm [16] or random forest algorithm [17]. 3) The Co-evolution-based approach where a pair of domains is regarded as interacting with each other if they share very similar phylogenetic trees [18]. However, one of the caveats for these DDI prediction approaches is that the information regarding the sequences and structures of these domains is neglected and as a result they suffer from low sensitivities and specificities.

It is known that segments of n contiguous amino acids (or n-grams) correlate to specific secondary structure elements [19,20]. Therefore, n-gram-based methods are widely exploited to predict the secondary structure or subcellular localization of proteins and to classify protein families using machine learning techniques [21-23]. The finding that n-grams are closely related to the secondary structure of protein domains prompts us to wonder whether n-grams can interact with each other. In fact, several studies have reported the interaction between n-grams. For example, molecular interaction exists between Smurf1 WW2 domain and PPXY motifs of Smad1[24]. Src-homology 3 domain (SH3) binds to a PXXP peptide [25]. Therefore, we hypothesize that some over-represented gram-gram interactions mediate DDIs and thus PPIs. In this study, we introduced a novel DDI prediction approach based on the primary sequence of proteins, by extracting n-gram frequencies from the annotated domain and DDI data set in yeast. This approach adopted substantial expansion from a related study reported previously [26].

Our approach, called GAIA, improves on other prediction approaches. When tested against a gold-standard data set, GAIA achieves a true positive rate (sensitivity) of 82% with a false positive rate (1 – specificity) of 21% and performs more accurately when the length of the gram is set to 4 amino acids. Using GAIA, we generated a list of 4-gram pairs that are significantly over-represented in the DDI data set. We postulate that these pairs mediate the DDIs in yeast. Overall, we demonstrate that GAIA, a gram-based method, provides a novel and reliable way to predict DDIs that may mediate PPIs in yeast. Our results, which show the localization of interacting grams/hotspots, provide testable hypotheses for experimental validation. Complemented with other prediction methods, this study facilitates us to elucidate the entire interactome of cells.

## Results and discussion

### Performance of the GAIA algorithm

**Figure 1:**
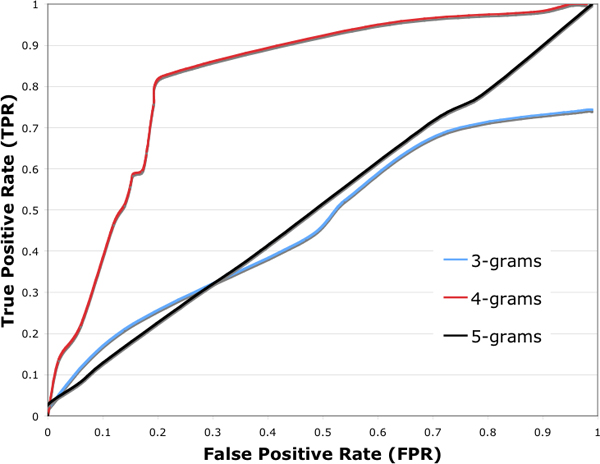
**The performance of the GAIA algorithm using different length gram pairs**. Curve of receiver operating characteristics (ROC) plotted for different thresholds when tested against the gold-standard positive and negative data set. The area under the curve plotted by 3-gram is 0.51, 0.79 for 4-grams and 0.52 for 5-grams, respectively.

To evaluate the performance of our algorithm, we tested the GAIA algorithm against both gold-standard positive and negative PPI data sets by setting the length of n-gram to 4 and the threshold of DDI's hits to 8.3. For the positive data set, 82% (886 out of 1080) of interacting domain pairs were successfully predicted and 18% of interacting domain pairs were detected to not interact with each other. For the negative data set, 21% (161 out of 767) of non-interacting domain pairs were incorrectly detected to interact with each other. These results indicate that our algorithm achieves a sensitivity of 82% and a specificity of 79%. A receiver operating characteristic (ROC) curve was plotted by measuring the sensitivity and specificity of GAIA tested against two gold-standard data sets at different cut-off values of DDI's hits (Fig. 1). The area under the curve (AUC) for the 4-gram is 0.79, which suggests that GAIA has a decent predictive performance.

Next, we tested whether our predicted DDIs could be utilized to predict PPIs. When there is at least one of our predicted DDIs existing between a pair of proteins, this pair of proteins is predicted as interacting with each other. For the positive data set, it was observed that 76% (452 out of 595) of interacting protein pairs were successfully predicted. For the negative data set, 25% (149 out of 595) of non-interacting protein pairs were incorrectly detected to interact with each other, reaching a sensitivity of 76% and a specificity of 75% when the threshold of DDI's hits is set to 8.3. These results demonstrate GAIA superiority to even *in vivo *experimental PPI identification approaches [1-6,8] as pointed out by several recent publications [26-28]. However, it should be noted that PPIs are predicted in GAIA under the assumption that interactions of given proteins are mediated by pairs of domains. Therefore, GAIA is not able to predict those PPIs mediated by amino acid segments outside of known interacting domains.

In order to investigate whether some gram pairs act as sequence signatures or markers of PPIs, we assigned a probability score to each gram pair (see method section) and compared the performance of GAIA with probability scores to that without probability scores. By using weighed gram pairs with probability scores, GAIA improved the sensitivity of DDI prediction from 68% to 82% and specificity from 66% to 79%. This improvement reflects the importance of highlighting gram-pairs that are over-represented in pairs of interacting domains but not in pairs of non-interacting domains, suggesting that these gram-pairs can act as sequence signatures.

### Parameters of the GAIA algorithm

The GAIA algorithm is solely based on protein sequence so no further information such as protein function or protein evolution information is needed. Only two parameters are needed to tune GAIA: (i) the length of gram (L_g_). Different gram lengths (3-grams, 4-grams, and 5-grams) have been tested. From observations of the ROC plots (Fig. 1), we found that with gram length of 3 or less, the DDI hits are not specific to the input DDI data set, therefore, yielding low true positive and high false positive rates. Conversely, with gram length of 5 or more, the DDI hits are too specific/low to differentiate between the positive and negative data sets. Therefore, we concluded that 4-gram yielded the best accuracy; and (ii) the threshold of the number of DDI hits (N_hit_). Choosing a proper threshold value optimizes the sensitivity at the expense of the specificity. For example, setting a lower threshold results in an increased sensitivity, at the expense of a decreased specificity. Similarly, a higher threshold results in a decreased sensitivity with an increase in specificity. Based on the ROC plots, it was found that GAIA achieves a sensitivity of 82% and specificity of 79% when the threshold is set to 8.3 (Fig. 1).

### Case studies on predicted DDIs

**Figure 2:**
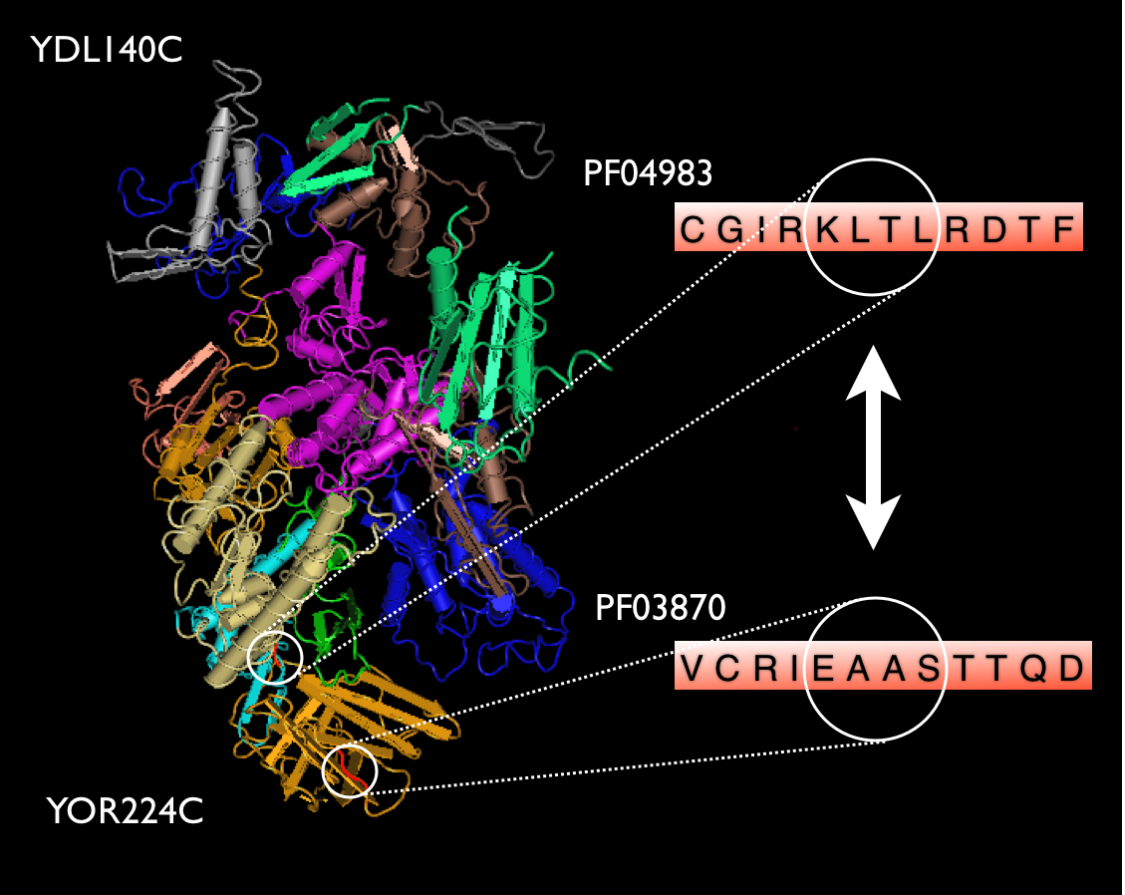
**3D structure of the interaction between RPB1/YDL140C and PRB2/YOR224C**. A 4-gram pair KLTL:EAAS (red region) that is predicted to contribute the DDI between PF04983 on RPB1 and PF03870 on RPB8 is highlighted. This gram pair is expanded on the right side of the figure for clarity. The figure was generated based on the PDB crystal structures (PDB: 1y1v) using the protein structural viewing tool Cn3D [42].

Our predictions were directly validated for some PPIs using documented three-dimensional structures available in the literature. For example, RPB1 (YDL140C, NP_010141.1) and PRB2 (YOR224C, NP_014867.1), two subunits of RNA polymerase II, are known to interact with each other [29]. Based on the iPfam annotation, these two proteins have three DDIs: PF04983 vs. PF03870; PF05000 vs. PF03870; PF04922 vs. PF03870. GAIA successfully predicted the interaction between this pair of proteins. Interestingly, we also found a 4-gram pair (KLTL:EAAS) which may contribute to the PPI. The first 4-gram, KLTL, is located in the region of residues 533 – 536 which corresponds to PF04983 (RNA polymerase Rpb1) on RPB1. EAAS is located in the region of residues 27 – 30, which corresponds to PF03870 (RNA polymerase Rpb8) on RPB8 (Figure 2).

**Figure 3:**
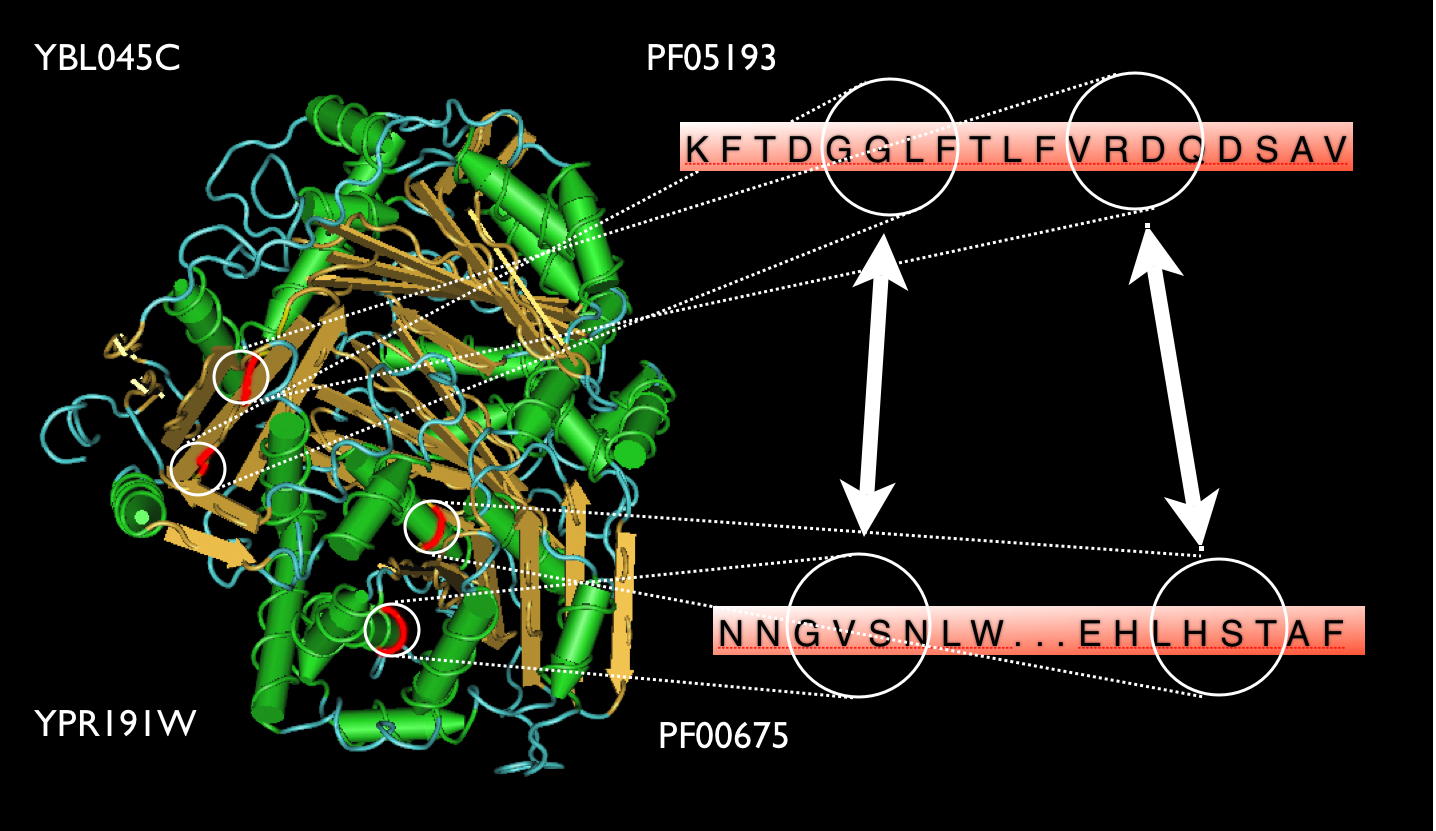
**3D structure of the interaction between COR1/YBL045C and QCR2/YPR191W**. Two 4-gram pairs GVSN:GGLF and LHST:VRDQ (red region) that are predicted to contribute the DDI between PF00675 on COR1 and PF05193 on QCR2 are highlighted. These gram pairs are expended on the right side of the figure for clarity. The figure was generated based on the PDB crystal structures (PDB: 1ezv) using the structural viewing tool Cn3D [42].

The interaction between COR1 (YBL045C, NP_009508.1) and QCR2 (YPR191W, NP_015517.1), two subunits of the ubiquinol cytochrome-c reductase complex (cytochrome bc1 complex) involved in cell respiration as a part of the mitochondrial inner membrane electron transport chain [30] was also examined. The interaction between COR1 and QCR2 has been validated by experimental approaches [1,5,29] and also by the GAIA algorithm. From the GAIA results, two gram pairs may contribute to this interaction. The first pair (GVSN:GGLF) is located in the region of residues 68 – 71 which corresponds to PF00675 (Peptidase family M16) on COR1 and the region of residues 282–285 which corresponds to PF05193 (Peptidase M16 inactive domain) on QCR2. The second pair (LHST:VRDQ) is located in the region of residues 164 – 167 which also corresponds to PF00675 on COR1 and the region of residues 289 – 292 which corresponds to PF05193 on QCR2 (Figure 3).

### Detecting new DDI-mediated PPIs and unknown domains

The GAIA tool performs well on previously reported PPIs mediated by DDIs in the gold-standard data set at a true positive rate of 82%. We therefore sought to apply the GAIA tool to identity novel PPIs and to determine the domains through which these interactions are mediated. Recently, Smy2p (YBR172C, NP_009731.2), a yeast gene encoding a protein of unknown function, was found to interact with Sec23p (YPR181C, NP_015507.1)/Sec24p (YIL109C, NP_012157.1) subcomplex and to participate in the coat protein complex II (COPII) vesicle formation from the endoplasmic reticulum (ER) [31]. The interaction between Smy2p and Sec23p was also predicted by GAIA. This successful prediction not only proves the ability of GAIA to detect novel PPIs but also suggests that the interaction might be mediated by DDIs. According to the domain annotations from the Pfam database [32], there is one annotated domain (PF02213: GYF) in Smy2p and 5 annotated domains (PF04810: zf-Sec23_Sec24; PF04811: Sec23_trunk; PF08033: Sec23_BS; PF04815: Sec23_helical; PF00626: Gelsolin) in Sec23p. Currently, there is no report of the DDIs between Smy2p and Sec23p in the literature. However, upon close examination of the prediction results from GAIA, we found two gram-pairs that may contribute to this PPI. The first pair has 18.7 DDI hits and is located at residues 410 – 413 of Sec23p, which corresponds to PF08033 and residues 68 – 71 of Smy2p. The second pair has 15.3 DDI hits and is located at residues 409 – 412 of Sec23p which corresponds to PF08033 and residues 499 – 502 of Smy2p. These results suggest that the Beta sandwich domain on Sec23p might well be involved in the PPI between Sec23p and Sym2p. Furthermore, we also found that another pair of 4-grams located at residues 616 – 619 in the Beta sandwich domain of Sec24p interacts with another 4-gram located at residue 713 – 716 of Sym2p, further supporting the important role for the Beta sandwich domain in the interaction between Sec23p/Sec24p and Sym2p. However, no known domain annotations have been associated with the location of the 4-grams on Smy2p, suggesting that potential domains of functional interest on Smy2p need to be further validated experimentally.

In addition to identifying new PPIs mediated by DDI, we also tested our GAIA tool on some protein pairs to infer new interacting domains from the predicted PPIs. Bud5 (YCR038C, NP_009967.2) and Bud8 (YLR353W, NP_013457.1) are two proteins involved in bud-site selection of diploid cells in yeast [33]. Krappmann *et. al *utilized the systematic structure-function analyses to identify that Bud5p physically interacts with Bud8p, and also interacts with Bud9p (YGR041W) which is involved in the delivery of the proteins to the cell poles [34]. They also found that the region of residues 74 – 216 on Bud8p and the region of residues 91 – 218 on Bud9p are interacting domains required to bind Bud5. Interestingly, GAIA also predicted a 4-gram pair that might mediate this interaction. This gram pair has 12.4 DDI hits and is located at residues 183 – 186 of Bud8p, which corresponds within the newly discovered 74 – 216 region mentioned above. This data supports our hypothesis that GAIA can be used to detect novel interacting domains from public domain-related data sets.

### Characterizing over-represented gram pairs

In our study, we have demonstrated that gram pairs are indeed valid elements in determining DDIs. In order to shed light on how these gram pairs actually interact with each other, we sought to identify and characterize the gram pairs over-represented in DDIs in the yeast proteome. We generated a list (Table 1) of over-represented gram pairs from the DDI data we used by quantifying their occurrences in both DDI data set and randomized negative data sets. The randomized negative data sets contain the same number of domain pairs as the iPfam DDI data set but these domain pairs do not exist in the iPfam DDI data set. As shown in Table 1, we found that most over-represented gram pairs are identical to each other. This finding suggests that some types of domains tend to interact with themselves. Such self-interactions could occur between SNARE transmembrane domains that promote the hemifusion-to-fusion transition [35]. Analyzing the DDI pair in iPfam, we found that such self-interactions between domains constitute approximately half (51%) of iPfam DDIs. It is therefore not surprising that these identical gram pairs occur so frequently in the DDI pairs. We also noticed that a majority of these interacting gram pairs consist of two consecutive hydrophilic (K, E or N) amino acids flanked by two hydrophobic amino acids (L, I or V), or two consecutive hydrophobic amino acids flanked by two hydrophilic amino acids. We reason that this kind of distribution of hydrophobicity may place the two amino acids in the middle in an environment where their hydrophobicity is reinforced by the surrounding amino acids of opposite hydrophobicity. Such reinforcement of hydrophobicity may increase the opportunity of this gram interacting with another gram with similar hydrophobicity reinforcement. This characteristic, however, does not exist in all of the over-represented gram pairs in our list, suggesting that other mechanisms also contribute to the interaction between gram pairs.

**Table 1:** A list of the most frequent gram pairs in DDI data set.

Gram A	Gram B	Frequency	P-value
LKEL	LKEL	36	2.2 × 10–16

ELLK	ELLK	35	7.7 × 10–16

LKKI	LKKI	33	2.2 × 10–16

LKKL	LKKL	32	2.2 × 10–16

LSKL	LSKL	32	2.2 × 10–16

DLSK	DLSK	31	2.2 × 10–16

ELLN	ELLN	31	2.2 × 10–16

LKSL	LKSL	31	2.2 × 10–16

EKLV	EKLV	30	2.2 × 10–16

LKNL	LKNL	30	2.2 × 10–16

For clarity, only gram pairs whose number of occurrence is greater than 30 were listed. A full list of gram pairs passing the cut-off value (Number of hits: 8.3) is provided online as a supplementary table. P-values for gram pairs were calculated using z-test by comparing the actual frequency of each gram pair to its corresponding frequencies in 1000 randomized domain-domain interaction data sets.

### Comparison between different approaches

DDI prediction algorithms similar to GAIA such as association method (AM) [14], maximum likelihood estimation approach (MLE) [15] and relative co-evolution of domain pairs approach (RCDP) [18] have recently been reported. It is difficult to compare the prediction accuracy of each approach directly because different testing datasets were utilized in each study. It is reported that AM achieves a sensitivity of 97% when tested against a small subset of interacting proteins. MLE achieved a sensitivity of 77.6% and a positive prediction value (PPV) of 42.5% when tested against a combined data set identified by yeast two-hybrid (Y2H) system. RCDP reported a sensitivity of 63.95% against a positive data set containing interacting proteins with DDIs derived from Protein Data Bank (PDB) crystal structures [18] and a specificity of 55.19% against a data set of randomly generated protein pairs. In order to eliminate the possibility that our gold standard data set is biased towards GAIA, therefore, we tested GAIA against the same testing data set (a combined data of two Y2H data sets derived from Uetz *et al. *[6] and Ito *et al. *[4]) used in each approach. GAIA achieved a PPV of 69% at the sensitivity is of 78% whereas AM and MLE achieved PPV of 42.5% and 24%, respectively, at the sensitivity of 78% [15], indicating that GAIA outperforms both AM and MLE. To account for the consideration that the improved performance is due to the better quality of input data, we also trained AM and MLE on 6304 PPIs containing identical number of DDIs as our GAIA training data set. We found that AM achieved a sensitivity of 51% with a specificity of 79% and MLE achieved a sensitivity of 57% with a specificity of 79% when tested against our gold-standard data set, proving that protein sequence information combined with structural information derived from iPfam is a better indicator to predict DDIs. In addition, GAIA also achieved a better sensitivity of 83% if the specificity was set to 55% in comparison to RCDP using the same testing data set as RCDP, illustrating that GAIA also performs better than RCDP. In summary, GAIA has the following advantages compared to other aforementioned approaches: 1) We have shown that GAIA can achieve better sensitivity and specificity in detecting DDIs; 2) GAIA is solely based on domain sequences and DDIs derived from PDB, rather than just PPI information, since prediction performance may be affected by poor PPI data set quality. We strongly believe that gram pairs such as those used in GAIA play a "signature" role in mediating the binding of a domain pair or protein pair. 3) By using protein sequences, GAIA precisely specifies the localization of interacting grams/hotspots.

## Conclusion

GAIA is a novel tool for identifying DDIs that mediate PPIs. GAIA takes the public DDI data set and the domain sequence data set as inputs and predicts the interaction between a query protein pair if the DDI hit frequencies of the gram pairs across the query proteins are above the preset threshold (8.3 DDIs). Tested against a "gold-standard" data set, GAIA achieves 82% true positive rate at the expense of 21% false positive rate. GAIA was used to identify a list of 4-gram pairs that is significantly over-represented in the DDI data set that may mediate PPIs. GAIA allows us to predict currently unknown interacting domains and to identify potential interacting gram pairs/hotspots between proteins. This study complements previous prediction approaches and also improves upon similar prediction modeling systems. The resultant predictions provide testable hypotheses for experimental validation. In the meantime, GAIA is limited by its highly intensive computational time (10 mins/per pair), which is currently being addressed by making changes to GAIA so that it can run in a distributed environment. While GAIA has good prediction capacity, increasing the size of the DDI data set would assist identification of a more complete set of gram pairs within the DDI data sets. This could ultimately lead us to a more complete identification of PPIs mediated by DDIs.

## Methods

The aim of this work is to predict DDIs based on the frequency of each possible gram-pair from a pair of query proteins. The frequencies of aforementioned gram-pairs are calculated from the annotated DDI data set and random data sets. In addition to predicting DDIs, GAIA also generates a list of gram pairs and their protein primary structure coordinates that contribute to the interaction between pairs of domains on query proteins. Details of how the GAIA algorithm works are provided in the following section, along with information about the data set collection, performance evaluation, and development environment.

### The GAIA algorithm

Step A. For each 4-gram *G*_*i*_, in query protein *A*, we generated a list of iPfam annotated domains *dlistG*[*i*] that contain this gram and the number of hits of this gram in each domain;

Step B. For each 4-gram *G*_*j *_appearing in query protein *B*, we also generated a list of Pfam annotated domains *dlistG*[*j*] that contain this gram and the number of hits of this gram in each domain;

Step C. For each gram-pair (*G*_*i*_, *G*_*j*_) between the query proteins *A *and *B*, we calculated the frequency of hits *freq*[*i*][*j*] for this gram-pair represented in interacting domain-domain pairs previously established in Pfam [36]. Then, the final frequency of hits *score*[*i*][*j*] for this gram-pair was weighted by *weightScore*[*i*][*j*] to determine if the number of its occurrences in the interacting domain pairs is statistically significant. The hit scores and weight scores are calculated by the following formulas:

(1) *hitscore*[*i*][*j*] = No. of hits *weightScore[*i*][*j*]

(2) *weightScore*[*i*][*j*] = P_(*real*|*random*)_(*Gram*[*i*][*j*])

Here, P_(real|random) _[i] [j] is the probability of the number of occurrences of *Gram*[*i*][*j*] in the interacting domain pairs is expected at random. Comparable control domain pairs were randomly generated by pairing domains from the DDI data set.

**Figure 4:**
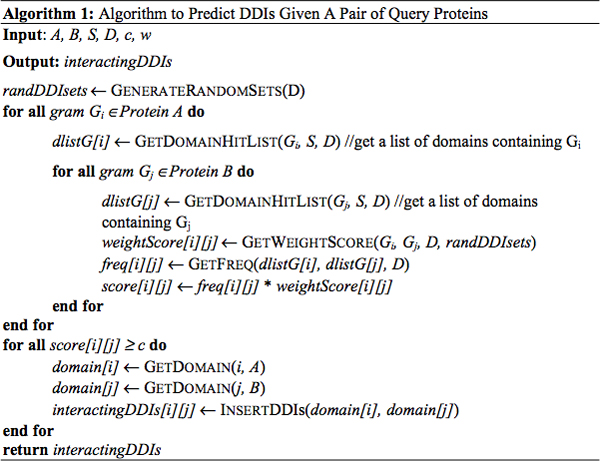
Algorithm to predict DDIs given a pair of query proteins.

Step D. For each gram-pair generated from Step C, if the hit frequency was over the preset threshold *c *and this gram-pair was located in a domain region, then this gram-pair and their corresponding domain pair was predicted to interact with each other. A profile containing the number of hits and the positions of the gram-pairs in the input query protein pair was simultaneously generated. This profile is important because it provides information on the amino acid hotspots that are potentially contributing to the physical interaction between the pair of query proteins (Fig. 4).

### Data set collection

We compiled 3,020 DDIs in yeast and their corresponding amino acid sequences from Pfam [32], a database containing protein domains and domain families, and iPfam [36], a database of DDIs derived from their RCSB Protein Data Bank (PDB) crystal structures [37]. For the purpose of evaluating prediction performance, we also used a "gold-standard" dataset that contained 595 PPIs compiled from a PPI dataset identified by the homologous protein interaction verification (HPIV) method [38]. It is reported that the HPIV positive dataset has better quality when used as the training data set for predicting PPIs [38]. All interacting protein pairs in our positive gold-standard dataset were expected to match three following criteria: 1) each pair is in the HPIV positive dataset; 2) each protein contains more than one domain; 3) each pair contains at least one iPfam domain-domain interaction. We also generated another "gold-standard" negative dataset containing 595 non-interacting protein pairs from the HPIV negative dataset. Compared to other simple approaches [39,40], HPIV applied a more sophisticated way to identify non-interacting protein pairs by multiple evidences such as functional, localization, expression and homology-based data [38].

### Evaluation of the GAIA algorithm

The performance of the scoring method was measured by the Receiver Operating Characteristic (ROC) curve and the Area Under the Curve (AUC). The area under the curve was calculated by the Wilcoxon rank sum test. ROC curve provides us an indicator of the sensitivity and how it is affected by the specificity. The area under the curve highlights discrimination (i.e., the ability of correctly classifying those interacting and non-interacting proteins). The ROC curve was generated by calculating the true positive rate (sensitivity) and the false positive rate (1-specificity) at the different thresholds on scores derived from PPIs and DDIs in the network, and combined scores from both kinds of interactions against the "gold-standard" data set. If the number of hits of any domain pair in a protein pair was above the threshold and it was in the DDIs of positive portion of the "gold-standard" data set, then it was regarded as a true positive. Alternatively, if it was not in the positive portion of the "gold-standard" dataset, then it was a false positive. If the number of hits of a domain pair in a protein pair was below the threshold and it was in the negative portion of the "gold-standard" data set, then it was regarded as a true negative. Alternatively, if it was not in the negative portion of the "gold-standard" data set, then it was a false negative.

The sensitivity, specificity and positive prediction value (PPV) were calculated as follows:

(3) $$Sensitivity=\frac{No.\;of\;True\;Positives}{No.\;of\;True\;Positives+No.\;of\;False\;Negatives}$$

(4) $$Specificity=\frac{No.\;of\;True\;Negatives}{No.\;of\;True\;Negatives+No.\;of\;False\;Positives}$$

(5) $$PPV=\frac{No.\;of\;True\;Positives}{No.\;of\;True\;Positives+No.\;of\;False\;Positives}$$

### Data and program availability

The related data sets and scripts, source code, and binaries are available for download from [41]. All scripts were written in Perl language version 5.8.6 and tested on a MacOS10.4.10 with a Macintosh work station (2.4 GHz Intel Core 2 Duo with 2GB 667 MHz DDR2 SDRAM). The source code and scripts are distributed under the terms of the Creative Commons Attribution License, which permits unrestricted use, distribution, and reproduction in any medium, provided the original author and source are credited.

## Competing interests

The authors declare that they have no competing interests.

## Authors' contributions

KXZ and BFFO conceived and designed the experiments. KXZ performed the experiments. KXZ analyzed the data. KXZ performed analysis and wrote the tools. KXZ and BFFO wrote the paper. All authors read and approved the final manuscript.
